# Clinical and genetic basis of congenital gonadotropin deficiency

**DOI:** 10.1093/hropen/hoag017

**Published:** 2026-03-15

**Authors:** Yi Wang, Jing Zhai, Imen Habibi, Zofia Kolesińska, Fernanda de Azevedo Correa, Yassine Zouaghi, Adelina Ameti, Alexia Boizot, Cecilia Perdices-López, Tommaso Todisco, Nicolas J Niederländer, James S Acierno, Andrea Messina, Federico Santoni, Nelly Pitteloud

**Affiliations:** Service of Endocrinology, Diabetology and Metabolism, CHUV, Lausanne, Switzerland; Faculty of Biology and Medicine, University of Lausanne, Lausanne, Switzerland; Department of Endocrinology, Genetics and Metabolism, Beijing Children’s Hospital, Capital Medical University, Beijing, China; Service of Endocrinology, Diabetology and Metabolism, CHUV, Lausanne, Switzerland; Faculty of Biology and Medicine, University of Lausanne, Lausanne, Switzerland; Service of Endocrinology, Diabetology and Metabolism, CHUV, Lausanne, Switzerland; Faculty of Biology and Medicine, University of Lausanne, Lausanne, Switzerland; Department of Pediatric Endocrinology and Rheumatology, Karol Jonscher Clinical Hospital, Poznan University of Medical Sciences, Poznan, Poland; Service of Endocrinology, Diabetology and Metabolism, CHUV, Lausanne, Switzerland; Faculty of Biology and Medicine, University of Lausanne, Lausanne, Switzerland; Service of Endocrinology, Diabetology and Metabolism, CHUV, Lausanne, Switzerland; Faculty of Biology and Medicine, University of Lausanne, Lausanne, Switzerland; Service of Endocrinology, Diabetology and Metabolism, CHUV, Lausanne, Switzerland; Faculty of Biology and Medicine, University of Lausanne, Lausanne, Switzerland; Service of Endocrinology, Diabetology and Metabolism, CHUV, Lausanne, Switzerland; Faculty of Biology and Medicine, University of Lausanne, Lausanne, Switzerland; Service of Endocrinology, Diabetology and Metabolism, CHUV, Lausanne, Switzerland; Faculty of Biology and Medicine, University of Lausanne, Lausanne, Switzerland; Service of Endocrinology, Diabetology and Metabolism, CHUV, Lausanne, Switzerland; Faculty of Biology and Medicine, University of Lausanne, Lausanne, Switzerland; Service of Endocrinology, Diabetology and Metabolism, CHUV, Lausanne, Switzerland; Faculty of Biology and Medicine, University of Lausanne, Lausanne, Switzerland; Service of Endocrinology, Diabetology and Metabolism, CHUV, Lausanne, Switzerland; Faculty of Biology and Medicine, University of Lausanne, Lausanne, Switzerland; Service of Endocrinology, Diabetology and Metabolism, CHUV, Lausanne, Switzerland; Faculty of Biology and Medicine, University of Lausanne, Lausanne, Switzerland; Service of Endocrinology, Diabetology and Metabolism, CHUV, Lausanne, Switzerland; Faculty of Biology and Medicine, University of Lausanne, Lausanne, Switzerland; Service of Endocrinology, Diabetology and Metabolism, CHUV, Lausanne, Switzerland; Faculty of Biology and Medicine, University of Lausanne, Lausanne, Switzerland

**Keywords:** gonadotropin deficiency, CHH known genes, CPHD known genes, anosmia, genetic overlap

## Abstract

**STUDY QUESTION:**

What is the clinical and genetic overlap across subtypes of congenital gonadotropin (Gn) deficiency?

**SUMMARY ANSWER:**

This study reveals substantial clinical and genetic overlap among Gn deficiency disorders, with shared genetic and developmental features across congenital hypogonadotropic hypogonadism (CHH), combined pituitary hormone deficiency (CPHD), and syndromic forms of Gn deficiency.

**WHAT IS KNOWN ALREADY:**

Congenital Gn deficiency includes a subset of hypogonadotropic hypogonadism (HH) and can result from defects at the level of the hypothalamus or the pituitary. It includes (i) CHH, further classified into normosmic CHH (nCHH) and Kallmann syndrome (KS); (ii) CPHD; and (iii) syndromic forms such as CHARGE syndrome and septo-optic dysplasia (SOD).

**STUDY DESIGN, SIZE, DURATION:**

The study included all probands with Gn deficiency recruited at a tertiary care center between 2011 and 2024 (n = 568), including 276 KS, 247 nCHH, 29 CPHD, and 16 syndromic Gn deficiency cases. All individuals underwent detailed clinical phenotyping followed by DNA sequencing.

**PARTICIPANTS/MATERIALS, SETTING, METHODS:**

Genetic analysis focused on pathogenic (P) and likely pathogenic (LP) variants and variants of uncertain significance (VUS) within established CHH and CPHD genes. Oligogenicity was assessed in the CHH/syndromic HH cohort (n = 523) compared with controls from 1000 Genomes (n = 601). Genetic overlap among CHH, CPHD, and syndromic Gn deficiency was systematically investigated.

**MAIN RESULTS AND THE ROLE OF CHANCE:**

Cleft lip/palate, dental agenesis, and ear abnormalities were recurrent across all Gn-deficient groups. Notably, some CPHD and SOD patients exhibited anosmia and a preserved Gn response to LH-releasing hormone (LHRH) stimulation, indicating a hypothalamic component to their HH. Rare variants in CHH genes were identified in 53% of KS probands (40% P/LP, 13% VUS) and 33% of nCHH probands (23% P/LP, 10% VUS). *FGFR1*, *ANOS1*, and *PROKR2* were most frequently mutated in KS, while *GNRHR*, *FGFR1*, and *KISS1R* predominated in nCHH. Oligogenic inheritance was detected in 15% of CHH cases, with variants in *FGFR1* being most commonly involved. Importantly, a substantial proportion (14%) of CHH patients without a molecular diagnosis carried rare variants predicted to be P or LP in genes typically associated with CPHD (e.g. *ROBO1*, *BRAF*, *FAT2*, and *DCHS2*). Conversely, several CHH-associated genes such as *FGFR1* and *FGF8*, already implicated in CPHD, were also identified in patients with CPHD and syndromic GN deficiency, further supporting a shared genetic architecture between CHH and CPHD.

**LARGE SCALE DATA:**

N/A

**LIMITATIONS, REASONS FOR CAUTION:**

Non-coding and copy number variants were not studied. Functional studies of the new candidate genes for CHH were not undertaken.

**WIDER IMPLICATIONS OF THE FINDINGS:**

This study highlights the importance of comprehensive clinical evaluation and broadened genetic testing in patients with Gn deficiency.

**STUDY FUNDING/COMPETING INTEREST(S):**

This work was supported by the Swiss National Foundation (NP) (Grant No. 310030B_201275 to N.P.) and the Natural Science Foundation of Beijing (Grant No. 7244338 to Y.W.). The authors declare no competing interests.

WHAT DOES THIS MEAN FOR PATIENTS? Congenital gonadotropin (Gn) deficiency is a rare cause of delayed or absent puberty and infertility, yet in many patients, the underlying cause remains unidentified despite extensive clinical evaluation. This study shows that conditions traditionally classified as distinct, such as congenital hypogonadotropic hypogonadism (CHH) and combined pituitary hormone deficiency (CPHD), often share overlapping clinical features and genetic causes. For patients, this means that a broader and more integrated diagnostic approach may be needed, including careful assessment of olfactory function, pituitary responses, and expanded genetic testing beyond classical disease categories. Recognizing this overlap may improve diagnostic accuracy, allow earlier identification of associated hormone deficiencies, and support more personalized clinical management and genetic counseling.

## Introduction

Congenital gonadotropin (Gn) deficiency, also referred to as hypogonadotropic hypogonadism (HH), is characterized by insufficient secretion of Gns, i.e. LH and FSH, from the gonadotrophs in the anterior pituitary, resulting in reduced sex steroid production and impaired gametogenesis. Gn deficiency represents a defining feature of isolated congenital hypogonadotropic hypogonadism (CHH), but it can also occur in combination with additional pituitary hormone deficiencies in the setting of combined pituitary hormone deficiency (CPHD) or as part of a recognized developmental syndrome with extra-pituitary manifestations. Such syndromic forms of Gn deficiency include CHARGE syndrome (coloboma, heart defects, atresia choanae, growth retardation, genital abnormalities, and ear abnormalities), septo-optic dysplasia (SOD), and holoprosencephaly (Kauvar and Muenke, 2010; Cerbone *et al.*, 2020).

CHH is caused by deficient secretion or action of GnRH, a hypothalamic neuropeptide that stimulates the release of Gns (LH and FSH) from the anterior pituitary. As a result, CHH presents clinically with partial or absent pubertal development and infertility (Boehm *et al.*, 2015). The estimated prevalence of CHH ranges from 1 to 10 per 100 000 live births (Fraietta *et al.*, 2013). Gn levels are low or inappropriately normal, i.e. normal Gn concentrations despite low serum testosterone or estradiol, which is physiologically inappropriate. Because serum GnRH cannot be reliably measured, congenital GnRH deficiency is inferred from the clinical presentation of absent or partial puberty, isolated Gn deficiency, and the absence of structural abnormalities on hypothalamic–pituitary MRI. CHH is further subclassified according to olfactory function into normosmic CHH (nCHH) and Kallmann syndrome (KS, i.e. CHH and anosmia). Developmental anomalies such as cleft lip and/or palate, dental agenesis, ear malformations, or hearing loss are frequently associated with CHH (Young *et al.*, 2019), although these features are not typically categorized within a defined syndromic entity. Genetically, CHH is highly heterogeneous, with over 60 genes identified to date; however, a molecular diagnosis is achieved in only ∼50% of patients (Cangiano *et al.*, 2021).

Congenital CPHD is a rare disorder typically defined by a deficiency of two or more pituitary hormones. Its global incidence is estimated at 1 per 8000 live births (Fang *et al.*, 2016). GN deficiency is present in 77–100% of CPHD cases (Rottembourg *et al.*, 2008; Guo *et al.*, 2013; Zheng *et al.*, 2017) and constitutes a major subgroup of interest in this study. The phenotypic spectrum of CPHD is broad, ranging from isolated hormonal deficiencies to complex syndromic forms. Notably, when CPHD is accompanied by craniofacial malformations or midline defects, a syndromic etiology such as SOD is likely. SOD is characterized by underdevelopment of the hypothalamic–pituitary axis, optic nerve hypoplasia, and midline brain anomalies, including agenesis of the septum pellucidum and/or corpus callosum (Sataite *et al.*, 2021). To date, more than 30 genes have been implicated in syndromic and non-syndromic forms of CPHD, with *HESX1*, *LHX3*, *LHX4*, *OTX2*, *POU1F1*, *PROP1*, *ROBO1*, and *SOX2* being the most extensively studied (Giordano, 2016; Dateki *et al.*, 2019; Misgar *et al.*, 2024). Despite these discoveries, the genetic etiology remains unresolved in ∼85% of CPHD cases (Giordano, 2016).

In this study, we aim to investigate the clinical and genetic overlap among a large cohort of patients with congenital Gn deficiency encompassing CHH, CPHD, and syndromic forms. Given the significant phenotypic and genetic heterogeneity of these conditions, we also explore the contribution of oligogenic inheritance to their pathogenesis.

## Materials and methods

### Subjects

Individuals with congenital Gn deficiency (CHH, CPHD with Gn deficiency, or with other syndromic forms associated with Gn deficiency) were enrolled at Lausanne University Hospital between 2011 and 2024. The study was approved by the Ethics Committee of the center, and all participants (or their guardians) provided written informed consent prior to inclusion.

### Clinical definitions and data collection

CHH was defined according to established criteria as follows: (i) absent or incomplete pubertal development by age 18 years, with or without micropenis and/or cryptorchidism; (ii) Gn levels are low or inappropriately normal, i.e. normal Gn concentrations despite low serum testosterone or estradiol, which is physiologically inappropriate; (iii) normal secretion of other pituitary hormones; and (iv) absence of structural anomalies in the hypothalamic–pituitary region on MRI (Pitteloud *et al.*, 2002; Cassatella *et al.*, 2018). Olfactory function was assessed by self-report, olfactory bulb imaging, and/or formal testing using the University of Pennsylvania Smell Identification Test (UPSIT). Patients with anosmia or hyposmia were classified as having KS.

CPHD was defined by deficiencies of at least two anterior pituitary hormones: growth hormone (GH), adrenocorticotropic hormone (ACTH), thyroid-stimulating hormone (TSH), and/or LH/FSH. For the purpose of this study, CPHD with Gn deficiency was defined as LH/FSH deficiency in combination with at least one additional pituitary hormone deficiency (Fang *et al.*, 2016).

Syndromic Gn deficiency included individuals meeting established diagnostic criteria for specific developmental syndromes in which Gn deficiency is a recognized feature. CHARGE syndrome was diagnosed according to the 2016 clinical criteria (Hale *et al.*, 2016), where Gn deficiency represents a common feature. SOD was defined by the triad of (i) optic nerve hypoplasia, (ii) agenesis of the septum pellucidum and/or corpus callosum, and (iii) Gn deficiency with or without additional pituitary hormone deficits (Sataite *et al.*, 2021). Moebius syndrome was characterized by the underdevelopment of the facial (cranial nerve VII) and abducens (cranial nerve VI) nerves, diagnosed according to established clinical guidelines (Monawwer *et al.*, 2023).

For all patients, detailed clinical, biochemical, and neuroradiological data were collected, covering both reproductive and non-reproductive phenotypes. In addition to patients with CHH, olfactory assessment and MRI of the olfactory bulbs and pituitary region were also performed in patients with CPHD and syndromic forms. Hormonal evaluations were primarily conducted at the referring centers, following local diagnostic protocols. The LHRH (GnRH) stimulation test was performed using a single subcutaneous dose of gonadorelin 100 μg. Serum LH was measured at 0, 30, 60, and 90 min. Baseline and peak LH values were recorded. Pituitary imaging was systematically reviewed and classified as follows: (i) anterior pituitary: normal, hypoplastic, aplastic, or enlarged; (ii) posterior pituitary: normal, ectopic, or hypoplastic; and (iii) pituitary stalk: normal, thin, thickened, or absent.

### Genetic studies

All patients underwent whole-exome sequencing (WES) or whole-genome sequencing (WGS). Genomic DNA was extracted from peripheral blood leukocytes using the PureGene kit (Qiagen, Hilden, Germany) or from saliva samples when blood was unavailable. Sequencing was conducted at two facilities: BGI (2011–2018, 2021–2024) and Geneva (2019–2020), with BGI targeting 50× coverage and Geneva 100×. WGS achieved a minimum depth of 30×, using 100–150 bp paired-end reads. WES employed Agilent V2/V5 probes (Agilent, Santa Clara, CA, USA) for earlier batches and Twist Bioscience probes (Twist Bioscience, San Francisco, CA, USA) for later batches, with all sequencing performed on the HiSeq 2000 platform (Illumina, San Diego, CA, USA) with a mean depth exceeding 50×.

Raw sequence data (FASTQ files) were processed using an in-house bioinformatics pipeline implemented with the Sentieon DNASeq toolkit (v202112.05) following a GATK-compliant workflow. Reads were mapped to the GRCh38 human reference genome, and single-nucleotide variants, as well as insertions/deletions (indels) under 50 bp, were identified. Variants were annotated with minor allele frequencies (MAFs) from gnomAD (v4.1) (https://gnomad.broadinstitute.org) and evaluated for pathogenicity using multiple *in silico* prediction tools like CADD (v1.6), SpliceAI (v1.3), REVEL, and Alphamissense, facilitated through ANNOVAR (v2020-06-07). Annotated variants were subsequently visualized, prioritized, and filtered using GenMasterTable (Zhai *et al.*, 2025).

Variants passing GATK filters, excluding those with <20% read support or within segmental duplications, were further filtered by the following criteria: a minimum quality score of 50, coverage ≥8, nonsense (stop gain, frameshift, and acceptor–donor splice sites ±6 bp from an exon), missense, inframe indels (insertion–deletion variants), and variants with a probability higher than 0.8 of causing a splicing defect as determined by the SpliceAI algorithm (Zouaghi *et al.*, 2024). Variants with MAF <1% were considered potentially pathogenic for autosomal recessive genes, and those with MAF <0.01% for autosomal dominant or X-linked genes.

For CHH genes in CHH and CPHD genes in CPHD, variants passing these filters were further annotated and classified using Varsome (https://varsome.com), following the American College of Medical Genetics (ACMG) standards criteria for pathogenicity. Sanger sequencing was performed to evaluate segregation in available family members. We retained pathogenic/likely pathogenic (P/LP) variants as well as variants of uncertain significance (VUS). Although VUS represent an inconclusive category, their low frequency and *in silico* predictions suggest possible effects on protein function. Therefore, we considered them potential contributors to oligogenic inheritance.

Known CHH and CPHD genes are presented in Supplementary Tables S1, S2, and S3.

Known CHH genes (n = 66) (Supplementary Tables S1 and S3) were defined as those meeting at least one of the following criteria: (i) identified as CHH genes in the European consensus statement, (ii) listed as CHH-related genes in the OMIM database (https://omim.org), and/or (iii) supported by publications reporting loss-of-function variants in CHH patients in genes expressed in organs/tissues relevant for GnRH neuronal biology.

Known CPHD genes (n = 40) (Supplementary Tables S2 and S3) were defined as those meeting at least one of the following criteria: (i) identified as CPHD-related genes in the OMIM database and/or (ii) supported by publications reporting loss-of-function variants in CPHD patients in genes encoding transcription factor or signaling molecules involved in pituitary gland development. The overlapping genes between the known CHH and CPHD gene sets (n = 7) were *CHD7*, *FGF8*, *FGFR1*, *PROKR2*, *SMCHD1*, *SOX2*, and *WDR11* (Supplementary Table S3).

*CHD7* is currently the only well-established gene for CHARGE syndrome. Known SOD genes (n = 7) were defined as those meeting at least one of the following criteria: (i) identified as SOD-related genes in the OMIM database and/or (ii) supported by publications reporting loss-of-function variants in SOD patients in genes affecting the development of the optic nerve or corpus callosum (Supplementary Table S4). A table including candidate genes for HH with weak supporting evidence is also included (Supplementary Table S5).

In addition, we investigated rare variants in CPHD-associated genes in the CHH cohort, as well as rare variants in CHH genes in the CPHD cohort, using the same stringent population frequency filters and *in silico* pathogenicity criteria described above. To enrich for variants predicted to be pathogenic, we retained nonsense, frameshift, or splice-site variants, as well as missense variants meeting at least two of the following three pathogenicity thresholds: CADD > 25.6, REVEL ≥ 0.685, or AlphaMissense ≥ 0.787. We also retained missense variants meeting at least two out of three broader pathogenicity criteria suggestive of potential deleteriousness: CADD > 20, REVEL ≥ 0.5, or AlphaMissense ≥ 0.5.

CoverageMaster (v2.0) (Rapti *et al.*, 2022) was used to detect CNVs in CHH- and CPHD-associated genes. In brief, this program uses depth of coverage from WGS and compresses these data into a multiscale wavelet space. The output is then analyzed through an iterative Hidden Markov Model to detect insertions or deletions >50 bp at nucleotide scale resolution.

### Oligogenicity in CHH

We conducted an expanded gene pair analysis in a cohort of 523 unrelated CHH probands and 601 controls from the 1000 Genomes Project (https://www.internationalgenome.org/). We focused on combinations of rare variants MAF <0.1% in 41 OMIM-listed CHH genes and performed an enrichment analysis using Fisher’s exact test to compare the frequency of known CHH gene pairs in CHH probands versus controls.

### Statistical analyses

Statistical comparisons of clinical phenotypes across clinical entities were performed using ANOVA with a significance threshold of *P *< 0.05 (two-sided). For the oligogenicity analysis, two-sided Fisher’s exact test *P*-values and Bonferroni-adjusted *P*-values (*P*-adj) were calculated based on comparisons between CHH patients versus controls.

## Results

### Clinical overlap between CHH, CPHD, and syndromic forms of Gn deficiency

**Figure 1.**
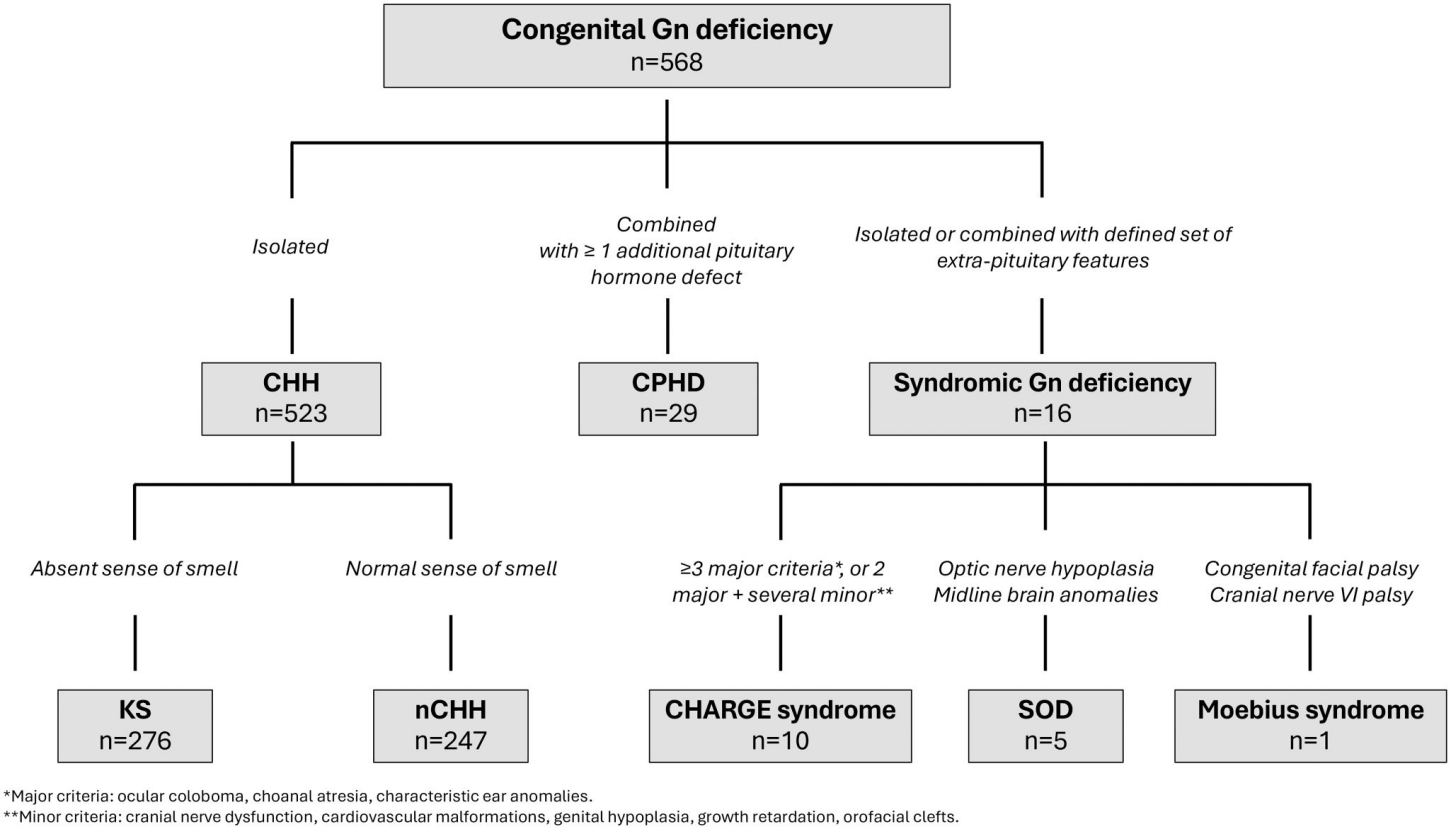
**Classification of patients with congenital gonadotropin (Gn) deficiency (n = 568)**. Patients were categorized according to pituitary hormone profile and extra-pituitary features into isolated CHH (n = 523), CPHD (n = 29), and syndromic Gn deficiency (n = 16). Isolated CHH included Kallmann syndrome (n = 276) and normosmic CHH (n = 247). Syndromic cases comprised CHARGE syndrome (n = 10), SOD (n = 5), and Moebius syndrome (n = 1). Gn, gonadotropin; CHH, congenital hypogonadotropic hypogonadism; nCHH, normosmic CHH; KS, Kallmann syndrome; CPHD, combined pituitary hormone deficiency; SOD, septo-optic dysplasia.

**Figure 2.**
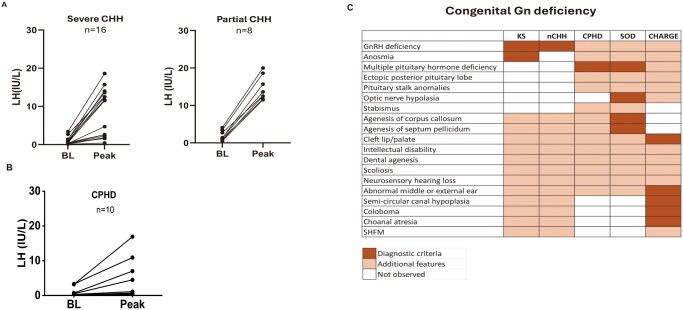
**Phenotypic overlap between CHH, CPHD, and syndromic Gn deficiency**. (**A** and **B**). LH response to GnRH stimulation in severe CHH, partial CHH and CPHD shows heterogeneous but overlapping peak LH values across groups. (**C**) Heatmap summarizing shared and syndrome-specific clinical features across CHH subtypes, CPHD, SOD, and CHARGE syndrome. CHH, congenital hypogonadotropic hypogonadism; CPHD, combined pituitary hormone deficiency; SOD, septo-optic dysplasia.

Our cohort of individuals with Gn deficiency (n = 568) was predominantly composed of patients with CHH, including 523 individuals: 276 diagnosed with KS and 247 with nCHH (Fig. 1). Additionally, we identified 29 probands with CPHD who exhibited GH deficiency (90%), TSH deficiency (52%), and ACTH deficiency (31%) in addition to Gn deficiency (Supplementary Fig. S1). Structural pituitary abnormalities included anterior pituitary hypoplasia or aplasia (65%), ectopic posterior pituitary in 39%, and pituitary stalk abnormalities in 43%. We also identified 16 patients with syndromic Gn deficiency (Fig. 1), including 10 individuals with CHARGE syndrome (9 presenting with CHH) and 5 patients with SOD, 3 of whom exhibited additional pituitary hormone deficiencies. Finally, one patient was diagnosed with Moebius syndrome, presenting with KS (Fig. 1). The majority of patients (71–100%, depending on subtype) were of European descent. The female-to-male ratio was ∼1:4 in the CHH group and 1:2 in CPHD. In line with the expected clinical presentation of Gn deficiency, 75% of patients across all diagnostic categories presented with absent puberty, whereas the remainder exhibited partial pubertal development (Table 1). As previously recognized, patients with severe CHH may show no response to an LHRH stimulation test (7/16), while all patients with partial CHH demonstrate a clear Gn rise (8/8) (Fig. 2A). Notably, 3 of the 10 CPHD patients (30%) with Gn deficiency who underwent LHRH testing exhibited a robust Gn response, with peak LH levels of 7.0, 10.9, and 16.9 IU/L, respectively (Fig. 2B). These responses indicate preserved pituitary gonadotroph function in this subset and support a predominantly hypothalamic origin of their Gn deficiency.

**Table 1.** Clinical phenotypes of patients with congenital Gn deficiency.

Phenotypes	CHH		CPHD (n = 29)	CHARGE syndrome (n = 10)	SOD (n = 5)
	KS (n = 276)	nCHH (n = 247)			
**Gender (female:male)**	54:222	73:174	7:16	3:7	1:4
**Reproductive phenotypes**					
Micropenis	45/90 (50%)	24/64 (38%)	8/13 (62%)	NA	NA
Cryptorchidism	30/38 (79%)*	12/26 (46%)	NA	NA	NA
Puberty (absent: partial)	60:16	43:23	13:5	6:1	3:1
Reversal	6/276 (2%)	7/247(3%)	0 (0)	0 (0)	0 (0)
**Non-reproductive phenotypes**					
Anosmia/hyposmia	276 (100%)	0 (0)	3/29 (10%)	7/10 (70%)	3/5 (60%)
Obesity	20/98 (20%)	17/82 (21%)	6/20 (30%)	2/6 (33%)	0/5 (0)
Synkinesia	12/276 (4%)	4/247 (2%)	0 (0)	1/10 (10%)	0 (0)
Cleft lip/palate	15/276 (5%)*	4/247 (2%)	1/29(3%)	2/10 (20%)	1/5 (20%)
Heart defect	4/276 (1%)	3/247(1%)	0 (0)	5/10 (50%)	0 (0)
Ear abnormalities	5/276 (2%)	3/247 (1%)	2/29 (7%)	6/10 (60%)	0 (0)
Hearing loss	18/276 (7%)	9/247 (4%)	1/29 (3%)	6/10 (60%)	2/5 (40%)
Dental agenesis	20/276 (7%)*	4/247 (2%)	1/29 (3%)	1/10 (10%)	0 (0)
Renal agenesis	5/276 (2%)	0 (0)	0 (0)	3/10 (30%)	0 (0)
Ataxia	3/276 (1%)	5/247 (2%)	1/29 (3%)	1/10 (10%)	0 (0)
Scoliosis	11/276 (4%)	11/247(4%)	0 (0)	1/10 (10%)	0 (0)
Clinodactyly	7/276 (3%)	3/247 (1%)	0 (0)	1/10 (10%)	0 (0)

* *P* < 0.05 for KS vs nCHH.

Gn, gonadotropin; CHH, congenital hypogonadotropic hypogonadism; nCHH, normosmic CHH; KS, Kallmann syndrome; CPHD, combined pituitary hormone deficiency; SOD, septo-optic dysplasia.

Non-reproductive anomalies, including cleft lip and/or palate (CLP), hearing loss, dental agenesis, and external ear malformations, were observed across all subgroups (Fig. 2C). Compared to nCHH, KS patients showed a significantly higher prevalence of cryptorchidism, CLP, and dental agenesis (*P* < 0.05), similar to that reported in a previous study (Dwyer *et al.*, 2023). Importantly, olfactory dysfunction, typically associated with KS, was also observed in other diagnostic categories. As expected, 100% of CHH patients in the KS subset reported anosmia or hyposmia. However, an impaired sense of smell was also identified in 7 of 10 (70%) patients with CHARGE syndrome and in 3 of 5 patients (60%) with SOD. Unexpectedly, 3 of 29 patients with CPHD (10%) also exhibited anosmia: a clinical feature typically associated with CHH. These findings indicate a broader spectrum of olfactory involvement across different forms of Gn deficiency than previously recognized, suggesting a potential shared developmental pathway.

### Complex genetic architecture of CHH

**Figure 3.**
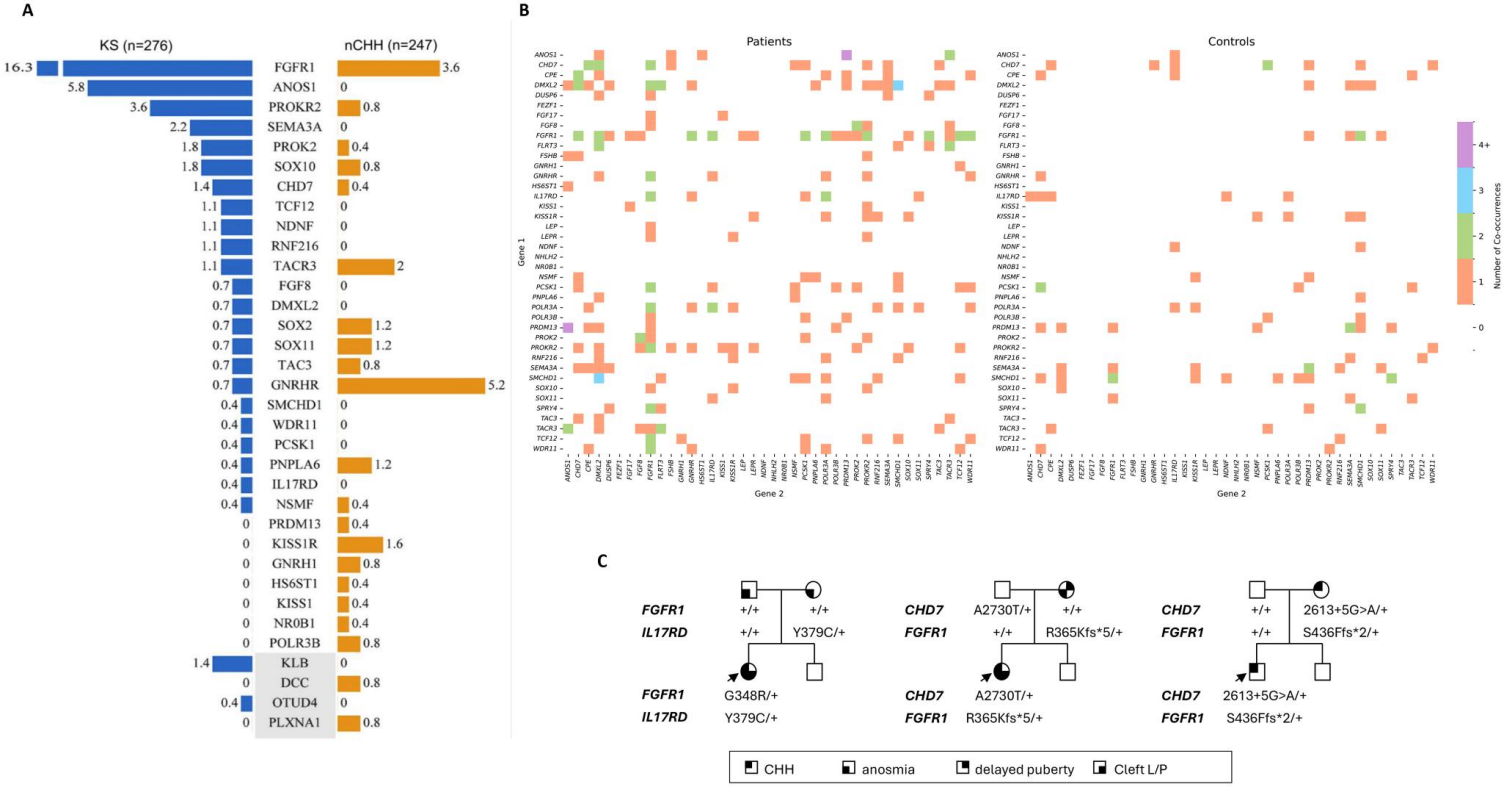
**Genetic architecture of CHH and CPHD in KS and nCHH patients**. (**A**) Frequency of rare variants in known CHH genes among probands with KS (blue) and normosmic CHH (nCHH, orange). Bars indicate the percentage of probands carrying variants in each gene. The overall variant-positive burden was higher in KS (53%) than in nCHH (33%). (**B**) Heatmap of gene–gene co-occurrence in CHH probands (left) and controls (right), restricted to OMIM-listed CHH genes. Each colored tile indicates the number of individuals carrying variants in both genes. (**C**) Representative pedigrees illustrating digenic inheritance patterns in CHH, including combinations of variants in *FGFR1*, *CHD7*, and *IL17RD*. Symbols indicate clinical features (CHH, anosmia, delayed puberty, cleft lip/palate). CHH, congenital hypogonadotropic hypogonadism; CPHD, combined pituitary hormone deficiency; KS, Kallmann syndrome; nCHH, normosmic CHH; OMIM, Online Mendelian Inheritance in Man; P/LP, pathogenic/likely pathogenic; VUS, variant of uncertain significance; AD, autosomal dominant; AR, autosomal recessive; MAF, minor allele frequency.

Rare variants in known CHH-associated genes were identified in 53% of KS patients (145/276), involving 25 of the 66 genes analyzed (Fig. 3A, Supplementary Tables S6 and S7). Among these variants, 40% were classified as P/LP, and 13% were categorized as VUS. The most frequently mutated genes in KS were *FGFR1* (16%), *ANOS1* (6%), and *PROKR2* (4%), and only two non-OMIM genes, *KLB* and *OTUD4*, were implicated (Fig. 3A). Among the 120 P/LP variants, 42% (n = 50) were novel, relative to gnomAD. Missense variants predominated (46%), followed by frameshift (26%) and stop–gain variants (17%). Additionally, three patients carried the same synonymous variant in *FGFR1* (p.A343A) (Supplementary Tables S6 and S7). This variant has previously been found to activate a cryptic splice donor site in Exon 8 of *FGFR1* (Courage *et al.*, 2019).

In the nCHH subgroup, 33% of patients (81/247) harbored rare variants in 21 of the 66 CHH genes (Fig. 3A, Supplementary Tables S6 and S7). Of these, 23% were P/LP and 10% were VUS. The mutational spectrum differed from KS, with the most frequently altered genes being *GNRHR* (5%), *FGFR1* (4%), and *KISS1R* (2%) (Fig. 3A). Two non-OMIM genes, *DCC* and *PLXNA1*, were identified in this group (Fig. 3A). Among the 74 P/LP variants, 47% (n = 35) were novel, including missense (46%), splicing (16%), and frameshift (14%) variants.

The rate of biallelic variants (either homozygous or compound heterozygous) was significantly higher in nCHH (11%) compared to KS (2%, *P* < 0.001), which is consistent with the fact that most recessive CHH genes predominantly underlie the nCHH phenotype.

In addition, two hemizygous CNVs were present in *ANOS1*, as well as one heterozygous CNV in *FGFR1* (Supplementary Fig. S2), in three unrelated KS patients: one 140 kb deletion began 33 kb upstream of *ANOS1* and extended through the first exon and intron of the gene and was detected; another 100 kb deletion was also observed and encompassed the last 11 of the 14 exons of *ANOS1* (Zouaghi *et al.*, 2024); an additional 4.9 kb heterozygous multi-exon deletion was detected in *FGFR1*, spanning the third exon of the gene to the last exon.

### High prevalence of oligogenicity in CHH

Using stringent filtering criteria (MAF <0.1% for all variants), we identified oligogenic combinations in 77 of 523 CHH probands (15%), involving 36 different CHH-associated genes (Fig. 3B). To evaluate the significance of this finding, we compared the frequency of oligogenicity in our CHH cohort (n = 523) with that in a control population from the 1000 Genomes Project (n = 601). Oligogenic events were significantly more prevalent in CHH patients than in controls, with *FGFR1* showing the strongest enrichment (adjusted P value = 0.0016). These results support the hypothesis that gene–gene interactions contribute substantially to disease pathogenesis in a significant proportion of CHH patients. To illustrate representative patterns of oligogenic inheritance, three pedigrees are shown in Fig. 3C.

### CPHD-associated genes mutated in CHH

**Figure 4.**
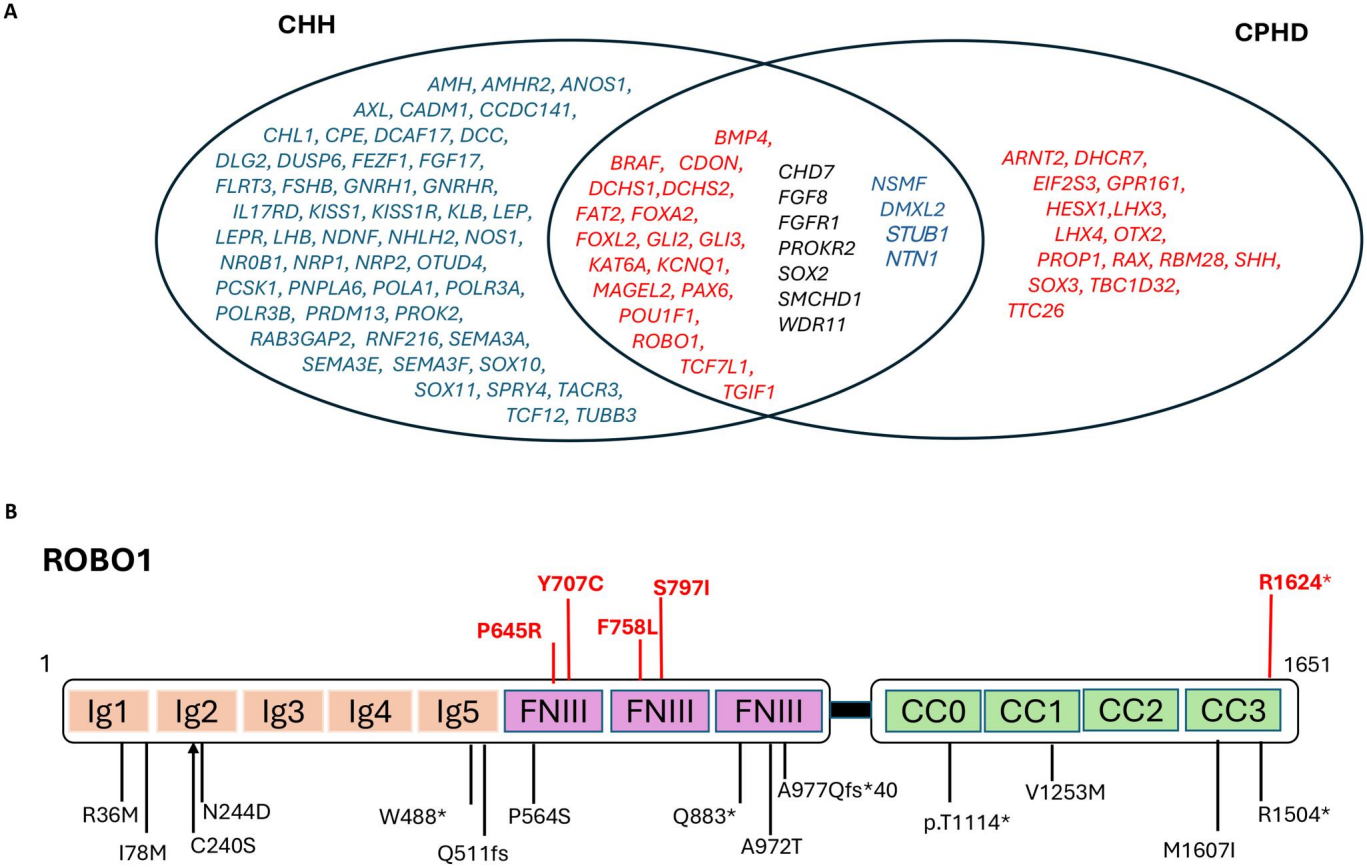
**Genetic overlap between CHH and CPHD and distribution of *ROBO1* variants**. (**A**) Venn diagram showing the overlap between genes implicated in CHH and CPHD. Genes in the intersection are shared between the two conditions; genes in red represent CHH genes identified in CPHD patients, and genes in blue represent CPHD genes identified in CHH patients. (**B**) Schematic of *ROBO1* with P/LP rare sequence variants (RSVs) mapped across Ig, FNIII, and CC domains. Variants found in our study are shown in red. CHH, congenital hypogonadotropic hypogonadism; CPHD, combined pituitary hormone deficiency; P/LP, pathogenic/likely pathogenic; Ig, immunoglobulin-like domain; FNIII, fibronectin type III; CC, cytoplasmic conserved motif.

Among CHH patients without identified mutations in known CHH genes, 14% (37/260, including 18 with KS and 19 with nCHH) harbored rare variants predicted to be P/LP in 18 genes typically associated with CPHD (Fig. 4A–B, Supplementary Table S8). Notably, 11 of these CPHD-associated genes encode proteins that physically interact with established CHH genes, highlighting shared molecular networks between pituitary and hypothalamic development (Supplementary Fig. S3). Furthermore, 11 of these genes (*BMP4*, *BRAF*, *CDON*, *DCHS1*, *GLI2*, *GLI3*, *KAT6A*, *MAGEL2*, *PAX6*, *ROBO1*, and *TCF7L1*) are expressed in developing GnRH neurons in mice (Zouaghi *et al.*, 2025). In human fetal brain transcriptomic datasets, *ROBO1* and *DCHS1* exhibit the highest expression levels in the human hypothalamus (Supplementary Table S9).

In the nCHH subgroup, the most frequently mutated CPHD-related genes were *ROBO1* (16%) including three rare missense variants (Fig. 4A and B, Supplementary Table S8), *FAT2* (16%) including two splicing variants (NM_001447: c.9194-5A>G and c.4790-4T>A), and *DCHS2* (11%) (Supplementary Table S8). We also identified a frameshift deletion in *PAX6* (c.721_732delinsG) and a frameshift variant in *POU1F1* (c.198delinsCATTTCTCG).

In the KS subgroup, the most affected CPHD-associated gene was *FAT2* (14%). Several loss-of-function variants were observed, including in *ROBO1* c.4570C>T, *FOXA2* (c.831_856delinsG), and *DCHS2* c.4095T>G, further highlighting the contribution of haploinsufficiency and truncating alleles in CPHD-associated pathways. Notably, two KS patients harbored a compound heterozygous mutation in *DCHS2* (NM_001358235: c.4124delinsGC with c.5456T, the other pair is c.2462T>C with c.2113G>A), consistent with a recessive mode of inheritance.

To compare the distribution of *ROBO1* variants identified in our CHH cohort with those previously reported in CPHD, we mapped all mutations onto the full-length ROBO1 protein (Fig. 4B, Supplementary Table S8). All CHH-associated variants identified in our study (shown in red) were located within regions critical for ligand binding and downstream signaling. Three missense variants, P609R, Y671C, and F722L, localized to the fibronectin type III (FNIII)1–2 region, which mediates SLIT ligand interaction and receptor dimerization. A fourth missense variant, S761I, mapped to FNIII2–3, whereas a truncating variant, R1524*, was positioned within the terminal CC3 motif, a key intracellular signaling domain. In contrast, CPHD-associated *ROBO1* variants reported in the literature (shown in black) were distributed across all domains of the protein. Collectively, these results demonstrate that several CPHD-associated genes, particularly *ROBO1*, *DCHS2*, and *FAT2*, may contribute to CHH pathogenesis. These findings highlight shared developmental pathways between hypothalamic and pituitary deficiencies and support a CHH–CPHD genetic continuum.

### Complex genetics for CPHD

Genetic screening of 40 CPHD-associated genes in the CPHD cohort identified P/LP variants in five genes and VUS in three genes, yielding a diagnostic rate of 20% (6/29 probands) (Supplementary Table S10). Among these, we detected a compound heterozygous variant in *PROP1*, two heterozygous deletion variants in *DCHS2*, and a frameshift variant in *GPR161*. We also identified P/LP variants in *FGFR1* and *FGF8*, two genes known to overlap CHH and CPHD phenotypes. Given the small number of affected individuals, potential oligogenic interactions could not be evaluated in this subgroup.

Additionally, we screened known CHH-associated genes in CPHD probands who did not harbor pathogenic variants in established CPHD genes. We identified five rare variants predicted to be pathogenic across three CHH genes (*NSMF*, *NTN1*, and *STUB1*) (Supplementary Table S11); three unrelated probands carried a heterozygous *NSMF* variant. These findings indicate that a subset of CPHD probands may carry contributory alleles within CHH-related pathways, supporting partial genetic overlap between the two conditions.

### Genetic heterogeneity of syndromes associated with Gn deficiency

Among the 16 syndromic Gn deficiency individuals, 10 met the 2016 clinical diagnostic criteria for CHARGE syndrome, each presenting with at least two major features (Figs 1 and 2C;  Supplementary Table S12). Notably, two patients displayed oligogenic inheritance patterns involving *CHD7* in combination with *IL17RD* or *NRP2*. Consistent with a prior study (Xu *et al.*, 2018), *CHD7* emerges as a shared genetic contributor, being mutated in nine CHARGE syndrome cases and seven CHH cases within our cohort (Supplementary Table S12).

Our cohort included six additional patients with Gn deficiency in the context of other syndromic conditions: five with SOD and one with Moebius syndrome (Supplementary Table S13). One SOD patient with CPHD carried a rare variant in *NTN1* (p.Asp39Gly); a second SOD patient with KS features had a pathogenic variant in *FGFR1* (c.828_828delinsAGG); and a third SOD/KS patient harbored LP variants in *CHD7* (p.Gly1982Glu) and *POLR3B* (p.X1076Arg). All variants identified in SOD patients occurred in genes classically associated with CHH, further reinforcing the developmental overlap between these disorders.

## Discussion

### Hypogonadism in CPHD: not necessarily of pituitary origin

Hypogonadism in CPHD has traditionally been attributed to intrinsic defects of the pituitary gland. Indeed, patients with CPHD frequently exhibit Gn deficiency associated with other pituitary hormone deficiencies, supporting a pituitary-based mechanism. However, accumulating clinical and experimental evidence indicates that the etiology of hypogonadism in CPHD is not exclusively limited to pituitary dysfunction. In our CPHD cohort, 3 out of 10 patients with Gn deficiency exhibited a normal response to the LHRH stimulation test, indicating that a sufficient number of functional gonadotrophs were preserved. An alternative, non-mutually exclusive explanation is that the primary defect resides at the level of GnRH neuronal development and function, supporting the dual-defect model involving both hypothalamic and pituitary contributions.

Further support for this dual origin comes from studies of *SOX2* deficiency in humans and mice. *SOX2* is a transcription factor essential for placode-specific development, and *SOX2* mutations are associated with a spectrum of midline and pituitary abnormalities. Individuals carrying *SOX2* mutations frequently present with hypoplastic pituitary glands, GH deficiency, and Gn deficiency: clinical features consistent with CPHD (Hietamaki *et al.*, 2022). Notably, *SOX2* mutations have also been identified in individuals with CHH (Cassin *et al.*, 2023). In *Sox2*-deficient mice, the gonadotroph defect is relatively mild, whereas there is a marked reduction in hypothalamic GnRH neurons. Consistently, two siblings with *SOX2*-related HH demonstrated a robust LH and FSH response to exogenous GnRH. Together, these human and mouse studies provide strong evidence for a dual (hypothalamic and pituitary) origin of HH in *SOX2* deficiency (Kelberman *et al.*, 2006; Jayakody *et al.*, 2012).

Further evidence comes from a recent cohort of 40 CPHD patients, in whom ∼60% demonstrated a normal LH/FSH response following 3 months of pulsatile GnRH treatment (Zheng *et al.*, 2017), again suggesting a hypothalamic component in the pathogenesis of hypogonadism. Similar findings emerged from our own earlier work in CHH, in which combined hypothalamic and pituitary defects were unmasked through pulsatile GnRH therapy (Sykiotis *et al.*, 2010b).

Collectively, these findings challenge the conventional dichotomy between CHH and CPHD, instead supporting the concept of a phenotypic and pathophysiologic spectrum involving variable contributions of both the hypothalamus and the pituitary. This distinction carries important clinical implications for fertility treatment. While CPHD patients are typically treated with exogenous Gns and CHH patients with pulsatile GnRH, identifying a hypothalamic or mixed (dual) origin of the defect suggests that pulsatile GnRH may also be effective in a subset of CPHD cases. Importantly, as the gonadotrophs require priming to respond to pulsatile GnRH, a diagnostic trial of pulsatile GnRH for 7 days (Pitteloud *et al.*, 2009) may be useful to accurately determine the level of dysfunction and guide optimal therapy for all patients with Gn deficiency.

### Anosmia in CPHD and syndromic Gn deficiency points toward a shared pathophysiological spectrum

In our cohort, anosmia or hyposmia was identified not only in KS but also in CPHD, in CHARGE syndrome and SOD, in association with rare variants in key developmental CHH genes such as *FGFR1* and *CHD7* (Table 1; Supplementary Table S6). These genes, implicated across phenotypes ranging from isolated anosmia (Kamarck *et al.*, 2024) to KS to complex syndromic presentations (Alkelai *et al.*, 2017), demonstrate marked pleiotropy. Notably, these findings reinforce the concept of overlapping genetic and developmental mechanisms across these conditions. A systematic assessment combining olfactory testing in Gn deficiency would be highly informative to pinpoint whether the defect is hypothalamic, pituitary, or dual in origin.

### Distinct genetic architecture of KS versus nCHH and insights into oligogenicity in CHH

The genetic yield of known CHH-associated genes in our CHH cohort is consistent with previous findings (Maione *et al.*, 2018). Consistently, the genetic architecture differs significantly between KS and nCHH, particularly in the distribution of the most frequently implicated genes and the prevalence of biallelic variants within the same loci. In addition, nearly half of the variants identified in known CHH genes had not been previously described, further expanding the mutational spectrum associated with this disorder.

Among the 194 P/LP variants detected, only one synonymous variant, *FGFR1* p. A343A, was observed across three patients. Although this variant is silent at the protein level, it has been reported to activate a cryptic splice donor site in Exon 8 of *FGFR1*, causing Hartsfield syndrome in two siblings with cryptorchidism and/or micropenis (Courage *et al.*, 2019). In our cohort, the two patients carrying this variant also presented with cleft lip and/or palate, although neither showed split hand/foot malformations.

Oligogenic inheritance in CHH has been recognized for over a decade. Consistent with earlier reports estimating oligogenicity in ∼6–15% of CHH cases (Sykiotis *et al.*, 2010a; Carrico *et al.*, 2024), our data revealed a 15% rate of oligogenic inheritance: significantly higher than in controls (*P* < 0.001). Among the genes contributing to oligogenic combinations, *FGFR1* was the most frequently involved, reaching statistical significance (*P*_adj_ = 0.01). Although enrichment analyses indicate that *FGFR1* contributes the strongest signal, our findings support a broader oligogenic contribution to CHH. Multi-hit interactions, particularly involving *FGFR1*, may influence phenotypic variability, but they still require careful interpretation, especially when VUS co-occur with pathogenic alleles.

### Shared genetic architecture between CHH and CPHD: a central role of *ROBO1* and protocadherin genes

We identified a substantial group of 18 additional genes, beyond the seven already known, that lie at the intersection of both phenotypes, indicating shared developmental pathways between hypothalamic GnRH neurons and pituitary organogenesis. Among CHH patients who tested negative for mutations in established CHH genes, these overlapping genes include key morphogens (*BMP4*, *FGF8*), transcription factors (*PAX6*, *SOX2*, *FOXA2*, *FOXL2*), and components of planar cell polarity and axon guidance (*DCHS1/2*, *FAT2*, *ROBO1*). Together, these findings support a convergent developmental vulnerability affecting both the hypothalamus and pituitary. Several of the shared genes (e.g. *KAT6A*, *KCNQ1*, *MAGEL2*) have also emerged in neurodevelopmental conditions (Chen *et al.*, 2020; Fallah *et al.*, 2020; Jiang *et al.*, 2020), further reinforcing the concept of pleiotropic developmental syndromes that may manifest clinically with CHH, CPHD, or a spectrum involving both. There were 260 (50%) CHH patients and 23 (79%) CPHD patients who had no relevant variants in known CHH or CPHD genes. When including CPHD-associated genes in CHH, the numbers were 223 (43%) for CHH and 18 (62%) for CPHD.

Within this group, *ROBO1*, a member of the Roundabout (Robo) family of axon guidance receptors, emerged as a compelling representative example of an overlapping gene, as multiple variants identified in CHH probands clustered across its FNIII domains. We identified five heterozygous RSVs in *ROBO1* among CHH patients. *ROBO1* encodes a transmembrane receptor featuring five immunoglobulin-like domains and a FNIII domain and it interacts with the extracellular ligand *SLIT2* to mediate axonal repulsion (Andrews *et al.*, 2006). Notably, several CHH-associated genes, such as *DCC*, *NDNF*, and *FLRT3*, also encode proteins containing evolutionarily conserved FNIII domains (Bouilly *et al.*, 2018; Messina *et al.*, 2020), underscoring the potential relevance of this structural motif in GnRH neuron development. The clinical relevance of *ROBO1* in pituitary and hypothalamic development has gained increasing support. In a cohort of patients with pituitary stalk interruption syndrome (PSIS), 20% (5/25) carried rare heterozygous *ROBO1* variants (Huang *et al.*, 2022). Another PSIS study identified one patient with HH and a *ROBO1* variant (Bashamboo *et al.*, 2017). In a larger study of congenital hypopituitarism, 5.8% (8/137) carried heterozygous RSV in *ROBO1* and presented with isolated GHD or CPHD (Martinez-Mayer *et al.*, 2024). Additionally, one KS patient was found to carry an intronic breakpoint in *SCEL* (Chromosome 13) together with an intergenic breakpoint between *ROBO1* and *ROBO2* (Chromosome 3); bioinformatic analysis implicated all three genes in neuronal migration and differentiation (Zhu *et al.*, 2020). Taking together with the established role of *ROBO1* in neural development, these findings implicate *ROBO1* in the pathogenesis of CHH.

Rare variants in *FAT2* and *DCHS2* were also identified in CHH patients lacking mutations in known CHH genes. These genes encode atypical protocadherins that function as ligand–receptor pairs, regulating planar cell polarity, cell adhesion, and tissue morphogenesis: processes critical for forebrain and pituitary development (Mao *et al.*, 2016). The presence of recurrent, truncating, and splice-disrupting variants in *DCHS2* and *FAT2* strongly supports their potential contribution to the CHH–CPHD developmental spectrum. Both genes encode atypical protocadherins that form a ligand–receptor pair and regulate planar cell polarity, cell adhesion, and tissue morphogenesis: processes central to forebrain and pituitary development. The accumulation of ultra-rare LP variants across multiple unrelated CHH patients, together with supportive data from PSIS and CPHD cohorts and from *Dchs2*^–/–^ and *Fat2*^–/–^ mouse models (Lodge *et al.*, 2020), strongly implicates disruption of this protocadherin signaling axis in hypothalamic–pituitary development and GnRH deficiency. These findings position *DCHS2* and *FAT2* as biologically plausible contributors to the shared genetic architecture linking CHH and CPHD.

Taken together, a working hypothesis is that CPHD caused by classical transcription factor defects involved in pituitary lineage specification reflects primary pituitary Gn deficiency, typically accompanied by structural pituitary abnormalities on MRI. In contrast, CPHD associated with CHH-type gene variants, especially those affecting neuronal migration, axon guidance, ciliogenesis, or hypothalamic patterning, may represent a hypothalamic GnRH disorder in which defective hypothalamic development or trophic input leads to secondary pituitary hormone deficits, even in the setting of a structurally normal pituitary. Moreover, genes such as *BMP4*, *FGF8*, and *FGFR1*, which are co-expressed in both the olfactory and pituitary placodes, provide a developmental link between CHH and CPHD. Disruption of these shared signaling pathways can simultaneously impair GnRH neuronal development and pituitary lineage specification, supporting a model in which hypothalamic and pituitary defects arise from perturbations of common upstream morphogenetic programs rather than anatomically isolated lesions.

## Conclusion

Our findings reveal substantial clinical and genetic overlap between CHH and CPHD, challenging the traditional view of these disorders as distinct entities. Differences in genetic architecture between KS and nCHH, together with the unexpected presence of anosmia in CPHD patients, point toward shared developmental mechanisms involving both the hypothalamus and the pituitary. The identification of rare variants in *ROBO1*, *BRAF*, *FAT2*, and *DCHS2* further supports this convergence, implicating pathways governing neuronal migration, planar cell polarity, and tissue morphogenesis in the pathogenesis of both CHH and CPHD. These insights highlight the value of systematic olfactory testing and diagnostic pulsatile GnRH trials in all patients with Gn deficiency, enabling more accurate identification of the level of dysfunction and ultimately guiding personalized therapy.

## Supplementary Material

hoag017_Supplementary_Data

## Data Availability

The datasets generated during and/or analyzed during the current study are available from the corresponding author upon reasonable request.
