# Supplementary material for: Clinical and genetic basis of congenital gonadotropin deficiency
Source: Hum Reprod Open. 2026 Mar 15;2026(2):hoag017. doi: 10.1093/hropen/hoag017 (PMC13005924; doi:10.1093/hropen/hoag017)
Supplement: hoag017_Supplementary_Data [file hoag017_supplementary_data.zip › Supplemental Figure S2.pdf]

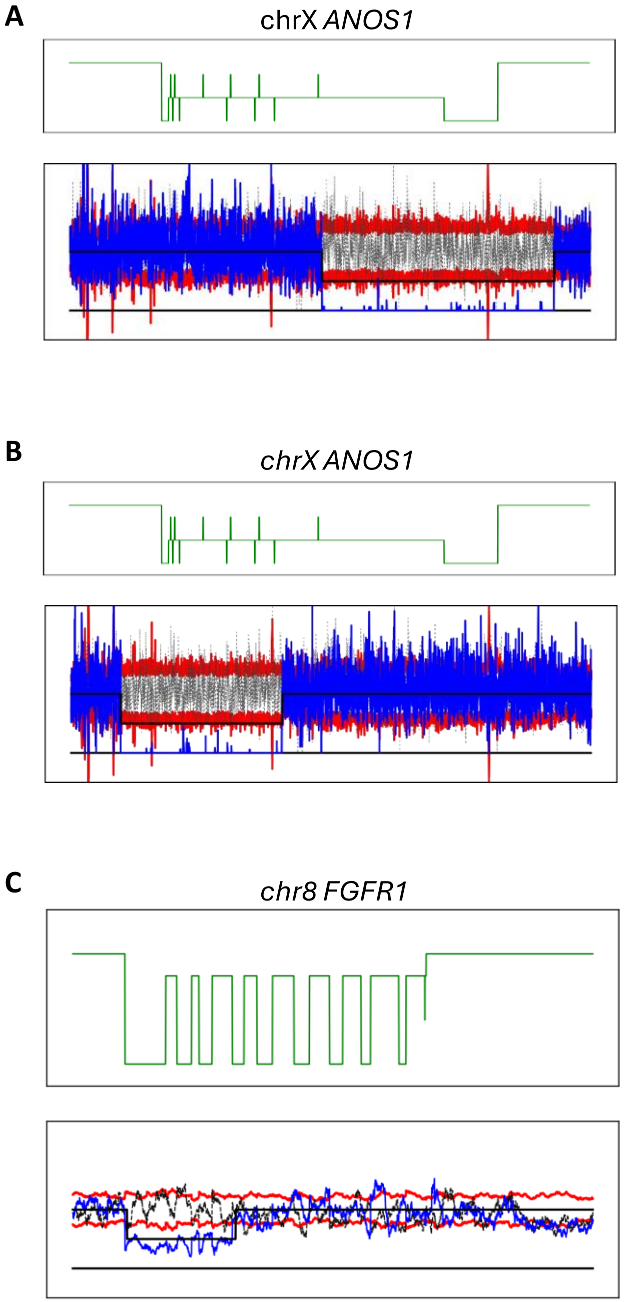

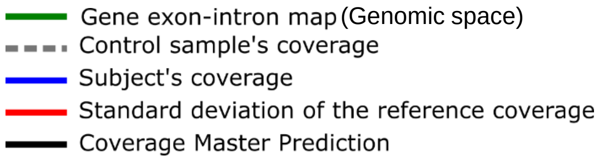

Supplementary Figure S2. **CNVs in *ANOS1* and *FGFR1*.** Two hemizygous CNVs were present in *ANOS1* and one heterozygous CNV in *FGFR1.* CNVs, copy number variants.
