# Supplementary material for: Clinical and genetic basis of congenital gonadotropin deficiency
Source: Hum Reprod Open. 2026 Mar 15;2026(2):hoag017. doi: 10.1093/hropen/hoag017 (PMC13005924; doi:10.1093/hropen/hoag017)
Supplement: hoag017_Supplementary_Data [file hoag017_supplementary_data.zip › Supplementary Figure S3.pdf]

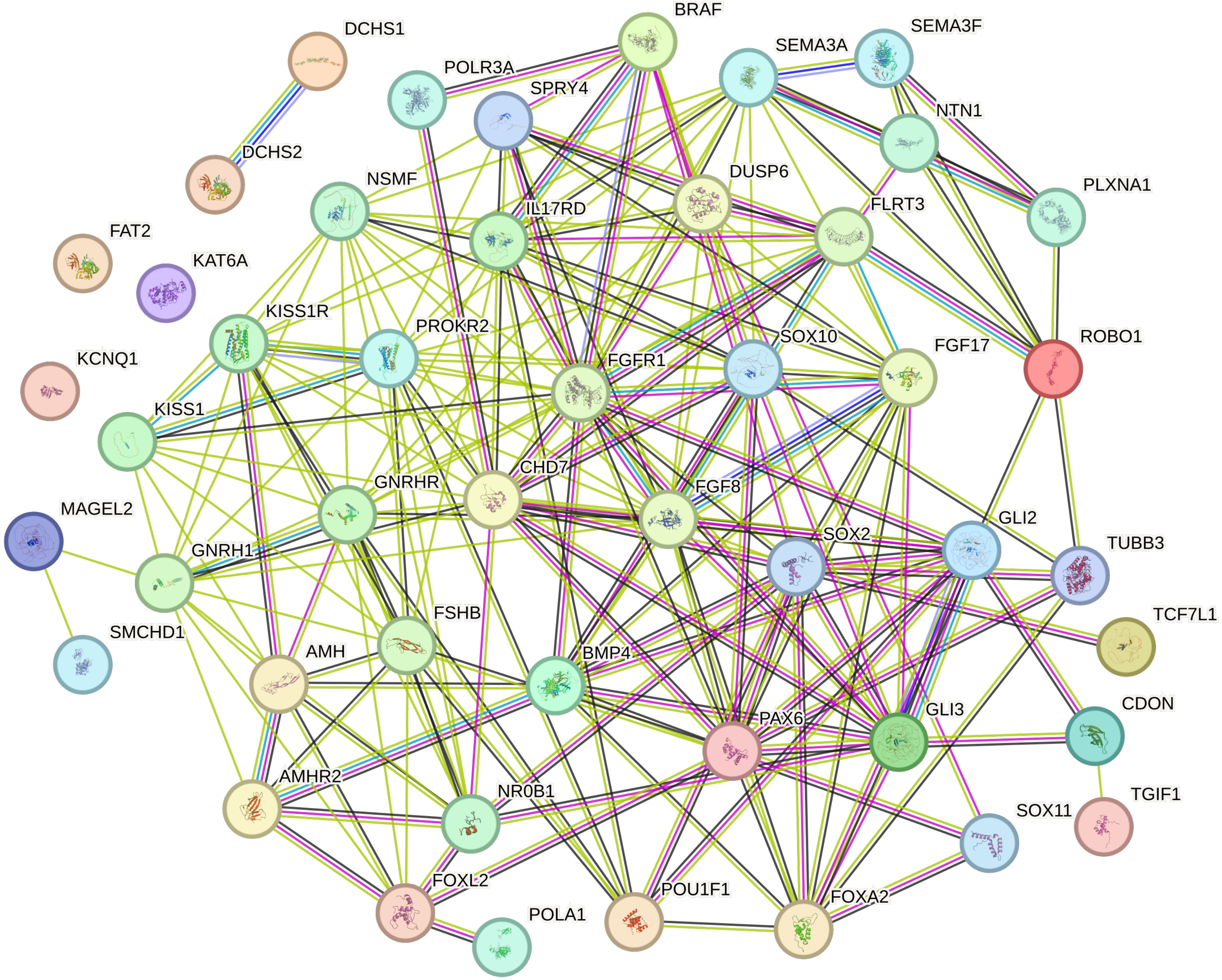

Supplementary Figure S3. **Interactions between CPHD genes and known CHH genes in CHH probands.** All CPHD genes were found in CHH probands without mutations in known CHH genes. CPHD, combined pituitary hormone deficiency; CHH, congenital hypogonadotropic hypogonadism.
