# Supplementary material for: Clinical and genetic basis of congenital gonadotropin deficiency
Source: Hum Reprod Open. 2026 Mar 15;2026(2):hoag017. doi: 10.1093/hropen/hoag017 (PMC13005924; doi:10.1093/hropen/hoag017)
Supplement: hoag017_Supplementary_Data [file hoag017_supplementary_data.zip › Supplemental Figure S1.pdf]

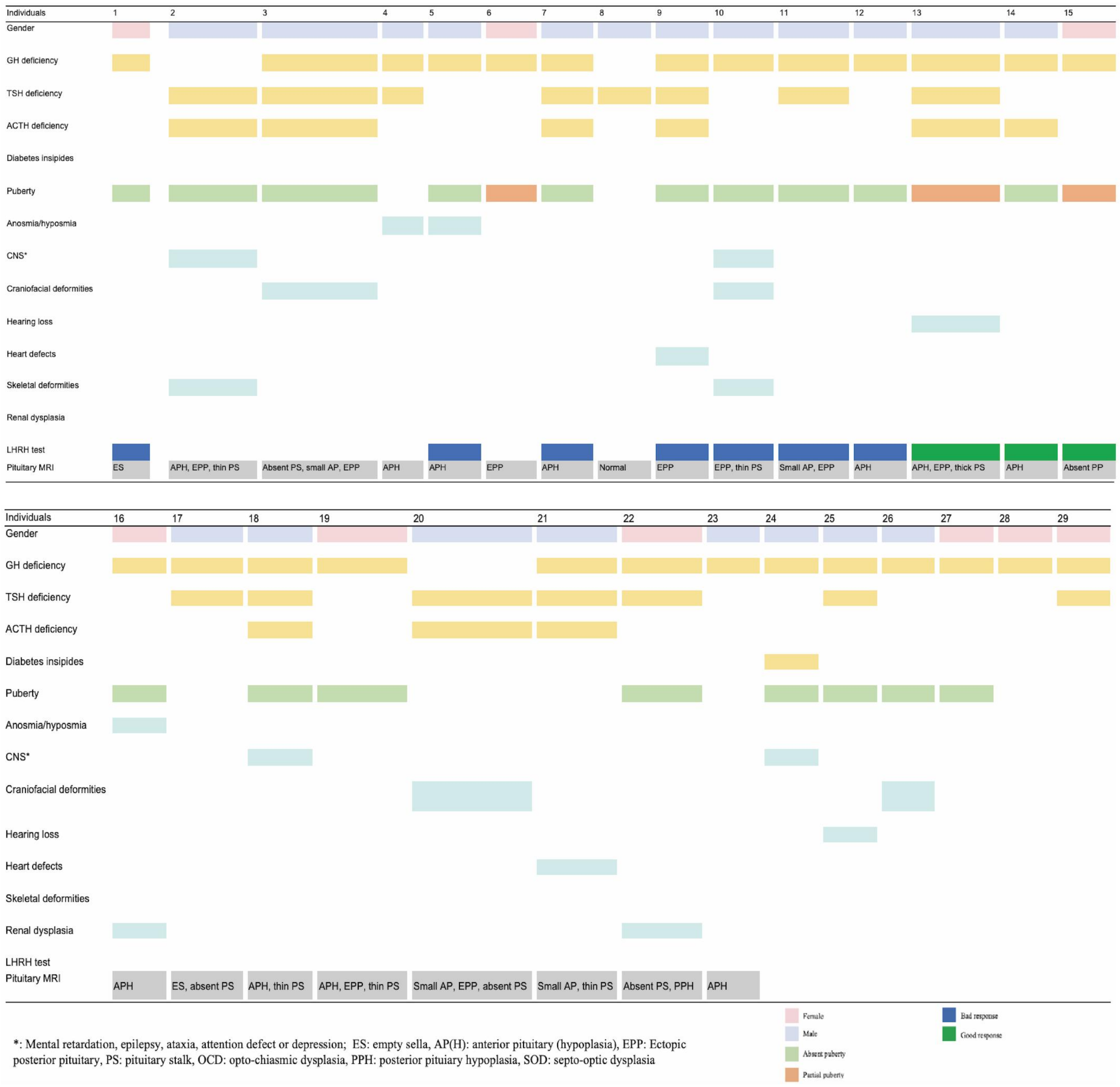

Supplementary Figure S1. **Clinical phenotypes in CPHD patients with Gn deficiency.**
GH: growth hormone; TSH: thyroid stimulating hormone; ACTH: adrenocorticotropic hormone; LHRH: luteinizing hormone – releasing hormone; AP: anterior pituitary; APH: anterior pituitary hypoplasia; EPP: ectopic posterior pituitary; ES: empty sella; PS: pituitary stalk; PPH: posterior pituitary hypoplasia; CPHD, combined pituitary hormone deficiency.
